# A mathematical determination of foveal attachment in primary rhegmatogenous retinal detachment when obscured by bullous retina

**DOI:** 10.1186/s40942-022-00359-3

**Published:** 2022-02-03

**Authors:** Obaid Kousha, Sonali Tarafdar, John Ellis

**Affiliations:** 1 Global Health Team, School of Medicine, Medical and Biological Sciences Building, North Haugh, St Andrews, KY16 9TF United Kingdom; 2 Department of Ophthalmology, Ninewells Hospital, James Arrott Drive, Dundee, DDY2 1SY United Kingdom

**Keywords:** Retinal detachment, Overhanging retina, Macula off

## Abstract

In primary rhegmatogenous retinal detachment (RRD), the foveal attachment is an important prognostic factors for post-operative vision. When the fovea is obscured by the RRD, its attachment status is considered uncertain. Using a model of the reduced emmetropic and − 10 dioptre myopic eye and the physical properties of the detached retina, we aimed to mathematically ascertain if it is clinically possible for the fovea to be attached while it is obscured by the primary RRD. With the patient upright, a primary RRD due to a 12 o’clock break directly above the fovea was considered. Mathematically, once the trough of the RRD touches the visual axis the edge of the RRD nearest to fovea is $2.77\,{\text{mm}}$ away from fovea in emmetropic eye and $2.89\;\,{\text{mm}}$ in myopic eye. When the RRD reaches the fovea, its trough is $2.20{\text{ mm}}$ below the visual axis in emmetropic eye and $2.29{\text{ mm}}$ in myopic eye. However, in vivo the RRD makes an acute angle with the retinal pigment epithelium and the corrugation of the retina in RRD shortens the retina. When these in vivo constraints are considered, in both of the above situations the fovea will be detached. If the fovea is obscured by an RRD, the fovea is very likely to be detached. In idiomatic terms, if the fovea cannot be seen, the fovea cannot see. This is an important clinical diagnosis for appropriate triage of the patient.

**Supplementary Information:**

The online version contains supplementary material available at 10.1186/s40942-022-00359-3.

## Introduction

**Fig. 1:**
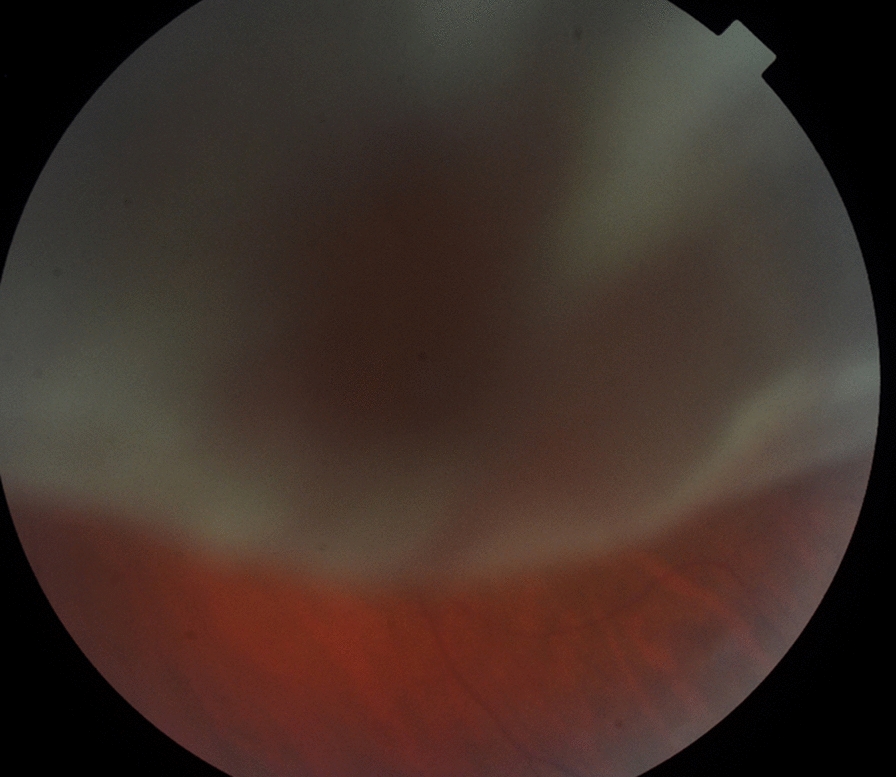
Primary RRD with detached retina ‘overhanging’ the fovea

A 58 years old man was referred from to the vitreoretinal service from a peripheral hospital with an acute primary rhegmatogenous retinal detachment (RRD) in the left eye. The gentleman could only see hand movement in the left eye and the referring clinician concluded that because the retina was ‘overhanging’ and obscuring the fovea as shown in Fig. 1, this could be a ‘macula-on’ RRD meriting expeditious surgical repair. The patient undergone 23 gauge 3 port pars plana vitrectomy within an hour of the image below (Fig. 1), but intraoperatively the fovea was found to be detached.

RRD is a common and potentially blinding condition with an incidence of approximately 1 in 10,000 of the population/year [1]. RRD develops due to a full thickness defect in the neurosensory retina. This results in ingress of fluid from the vitreous cavity through the defect which separates the neurosensory retina from the underlying retinal pigment epithelium (RPE) [2]. RRD is clinically divided into ‘macula-off’, where the fovea is detached, or ‘macula-on’, where the fovea is still attached [3]. Clinical importance of this dichotomisation is that visual outcome after a successful surgery is better in a ‘macula-on’ RRD before it progresses into a ‘macula-off’ RRD [4]. Therefore, this clinical distinction potentially confers extra urgency to ‘macula-on’ RRD to prevent it progressing into a ‘macula-off’ RRD.

Usually, clinical distinction between ‘macula-on’ RRD and ‘macula-off’ RRD is straightforward. However, sometimes the fovea is obscured by the detached retina ‘overhanging’ the fovea creating uncertainty regarding the status of the foveal attachment.

Using physical principles, we investigate whether it is possible to have an attached fovea when it is obscured by a detached retina in RRD.

## Methods

**Fig. 2:**
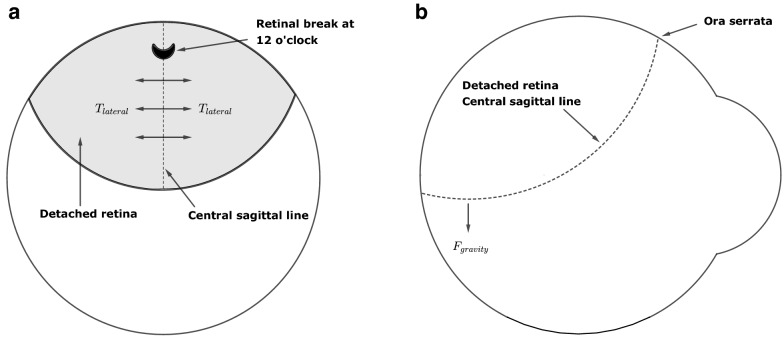
Physical forces acting on a freely hanging retina**.** Primary RRD due to a 12 o’clock break directly above the fovea hangs down like a hammock under the effect of gravity. As this ‘hammock’ is symmetrical, the resultant lateral forces, $T_{lateral}$, is zero along its central line (**a**). The central sagittal line can be assumed to hang under the effect of gravity, $F_{gravity}$, as a catenary (**b**)

We consider a clinical scenario there is a retinal break directly above the fovea causing an RRD as shown in the Fig. 2a. In this scenario the chance of an RRD ‘overhanging’ the fovea is maximised (Fig. 2b). The trough of the dotted central line (Fig. 2) is the lowest point of the hanging retina. The retina hangs under the effect of gravity like a hammock and as the RRD is symmetrical either side of the dotted central line, the resultant lateral force is zero on this line (Fig. 2a). Therefore, we can consider the dotted central line as a hanging cable under the effect of gravity—i.e. as a catenary (Fig. 2b).

A catenary is described by the hyperbolic cosine function:

$$y = a\cosh \left( \frac{x}{a} \right)$$

where $a$ depends on the horizontal tension along the cable and the weight of the cable per unit length. Therefore, the value of $a$ will be constant in the scenarios that we consider here.

Furthermore, we know that the superior ora serrata is approximately $120^\circ$ from the fovea and the inner diameter of the globe is $22 \,{\text{mm}}$ in an emmetropic eye [5]. We also considered a − 10 dioptre myopic eye with axial length of $27.13\, {\text{mm}}$ [6].

When considering an idealised model of the eye, we make the following assumptions:

1. The patient is examined upright
2. The vitreous is fully synchytic and syneretic and has the same viscosity and density as water
3. The RRD has a fully stretched smooth surface freely hanging under the effect of gravity.

In vivo, this model will be modified by corrugation of the detached retina reducing its arclength (Fig. 4b). The extent to which this happens will be hard to universalise but will be unlikely ever to be entirely non-existent.

## Results

**Fig. 3:**
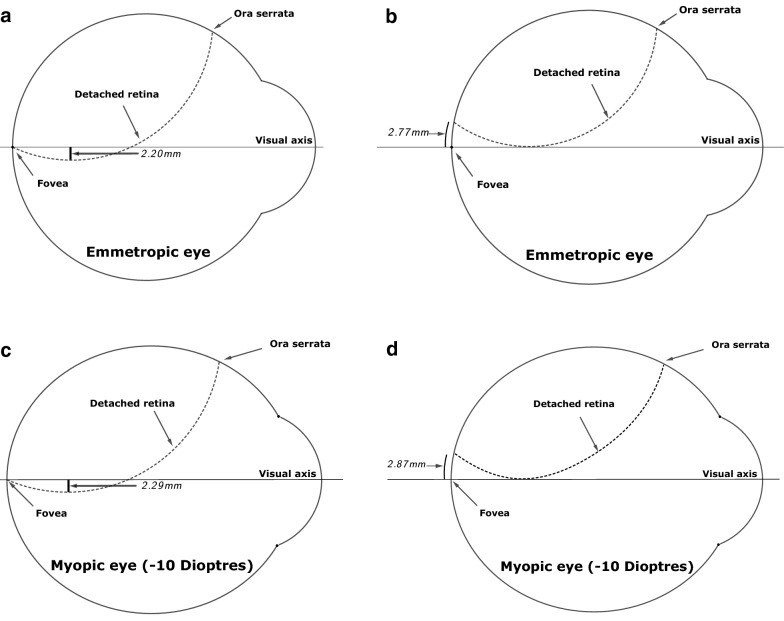
Foveal attachment status in primary RRD of different extent secondary to a 12 o’clock break. The vertical distance between the trough of the RRD and the visual axis when the posterior extent of the RRD is at the fovea for an emmetropic eye (**a**) and a − 10 dioptres myopic eye (**c**) in a mathematical model. The posterior extent of the RRD when the visual axis makes a tangent to trough of the RRD for an emmetropic eye (**b**) and a − 10 dioptres myopic eye (**d**) in a mathematical model

**Fig. 4:**
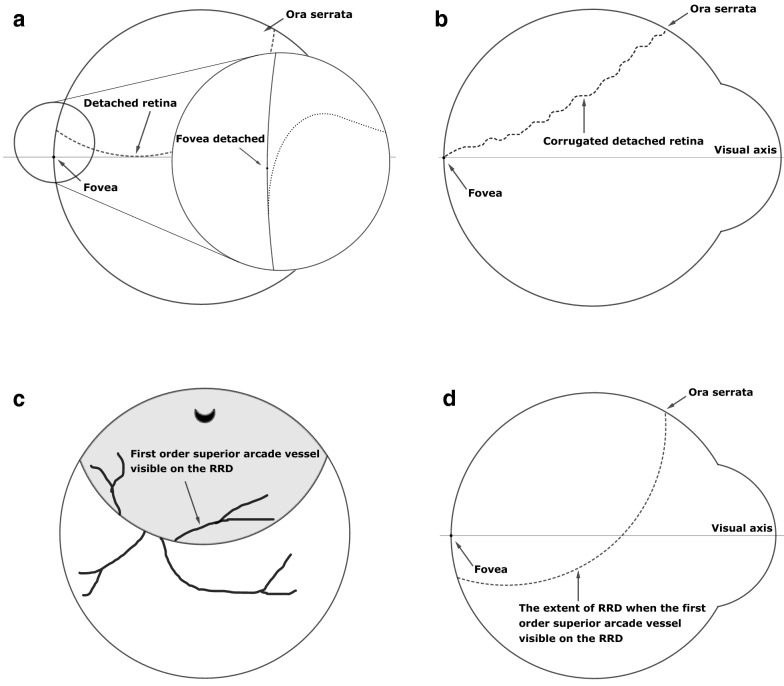
Foveal attachment status in primary RRD of different extent secondary to a 12 o’clock break. Clinically, the fovea is likely to be detached if the trough of the RRD makes a tangent to the visual axis as the RRD meets the RPE in an acute angle (**a**). Clinically, the corrugation of the retina can shorten the retina sufficiently to reveal the detached fovea, even if the posterior extend of the RRD is at the fovea (**b**). If the first order supero-temporal arcade vessel is visible on the RRD (**c**), the fovea is detached even in the mathematical model (**d**)

Using the assumptions above, we can calculate the following:

- The vertical distance between the visual axis and the RRD trough below the visual axis, when the edge of the RRD is at the fovea, is $2.20 \;{\text{mm}}$ for an emmetropic eye (Fig. 3a) and $2.29 \,{\text{mm}}$ for a − 10 dioptre myopic eye.
- The shortest distance between the fovea and the edge of the RRD when visual axis makes a tangent with lowest point of the RRD is $2.77\; {\text{mm}}$ or $14.4^\circ$ for an emmetropic eye (Fig. 3b) and $2.87\; {\text{mm}}$ for a − 10 dioptre myopic eye (Fig. 3d).
- In emmetropic eye if the first order superior arcade vessel is seen on the RRD, by definition, the fovea is off (Fig. 4c and d). Even if the vessels were at the trough of the RRD, the inferior edge of the RRD is calculated to be $8.8^\circ$ or $1.7\; {\text{mm}}$ below the fovea—i.e. the fovea is detached.

The calculation details have been described in Additional file 1.

## Discussion

Usually, RRD makes a shallow angle ($< 90^\circ$) as it meets the RPE. The models in Fig. 3 assume far larger angles which are not realistic. Hence, in vivo, it is not inconceivable that the fovea is already detached even in the scenario shown in Fig. 3a. Although with aid of optical coherence tomography (OCT) it has been shown that at the macula RRD can potentially make large angles with the RPE [7], this has only been demonstrated at the peripapillary area and not at the fovea or parafoveal area. In a recent study with the aid of OCT, it was found that in all patients where the ophthalmologist concluded that the RRD edge was at the level of fovea on clinical examination, the fovea was detached [8]. In all these cases, the OCT demonstrated a shallow RD extending beyond the perceived edge as demonstrated in Fig. 4a [8]. Furthermore, it was demonstrated that the OCT determined edge of the RRD was up to $2.5 \;{\text{mm}}$ below the clinically determined edge of the RRD [8].

In the idealised mathematical models, it seems that it is physically possible for the RRD to obscure an attached fovea. However, in clinical practice, the retina in RRD is never smooth, but it is corrugated making it shorter than the full length of smoothly stretched retina assumed by the model (Fig. 4b). Consequently, any corrugation of the detached retina (Fig. 4b) will shorten the arclength of the hanging retina compared with the scenario shown in Fig. 3a and c. If this shortening is sufficient to lift the lowest point of the RRD by more than $2.20 \;{\text{mm}}$ or $2.29\; {\text{mm}}$ shown in Fig. 3a and c, the detached fovea will be revealed. However, the length of the retina needs to reduce by a least a third to achieve this.

The elasticity of the retina might be considered as a factor in stretching the retina. However, give the specific gravity of the subretinal fluid in an acute RRD (approximately $1.02 \;{\text{g}}/{\text{cm}}^{3}$) [9], the retina ($1.0425 \;{\text{g}}/{\text{cm}}^{3}$) [10], and the vitreous ($1.0053{-} 1.008 \;{\text{g}}/{\text{cm}}^{3}$) (11), we believe there will be a minimum, if any, stretching of the retina due to gravity.

Moreover, the detached retina rarely makes a large angle with the RPE. In this regard, the assumptions of the model are rather generous. In vivo, even if the gravitationally lowest point of the RRD appears precisely on the visual axis (Fig. 3b and d), the fovea is likely to be detached (Fig. 4a).

## Conclusions

In summary, if the fovea is obscured by an RRD in both emmetropic and myopic eyes, it is likely that the fovea is detached and it is a ‘macula-off’ RRD. The clinical implication of correctly differentiating between a ‘macula-on’ RRD and a ‘macula-off’ RRD is that the patient could be triaged appropriately.

## Supplementary Information

**Additional file 1.** A mathematical model of primary rhegmatogenous retinal detachment secondary to a 12 o’clock break.

## Data Availability

Not applicable.
