# Supplementary material for: A mathematical determination of foveal attachment in primary rhegmatogenous retinal detachment when obscured by bullous retina
Source: Int J Retina Vitreous. 2022 Feb 3;8:10. doi: 10.1186/s40942-022-00359-3 (PMC8811976; doi:10.1186/s40942-022-00359-3)
Supplement: Supplementary file 1 — Additional file 1. A mathematical model of primary rhegmatogenous retinal detachment secondary to a 12 o’clock break. [file 40942_2022_359_MOESM1_ESM.pdf]

SUPPLEMENTARY MATERIAL

Mathematics of a catenary:

Figure S1:

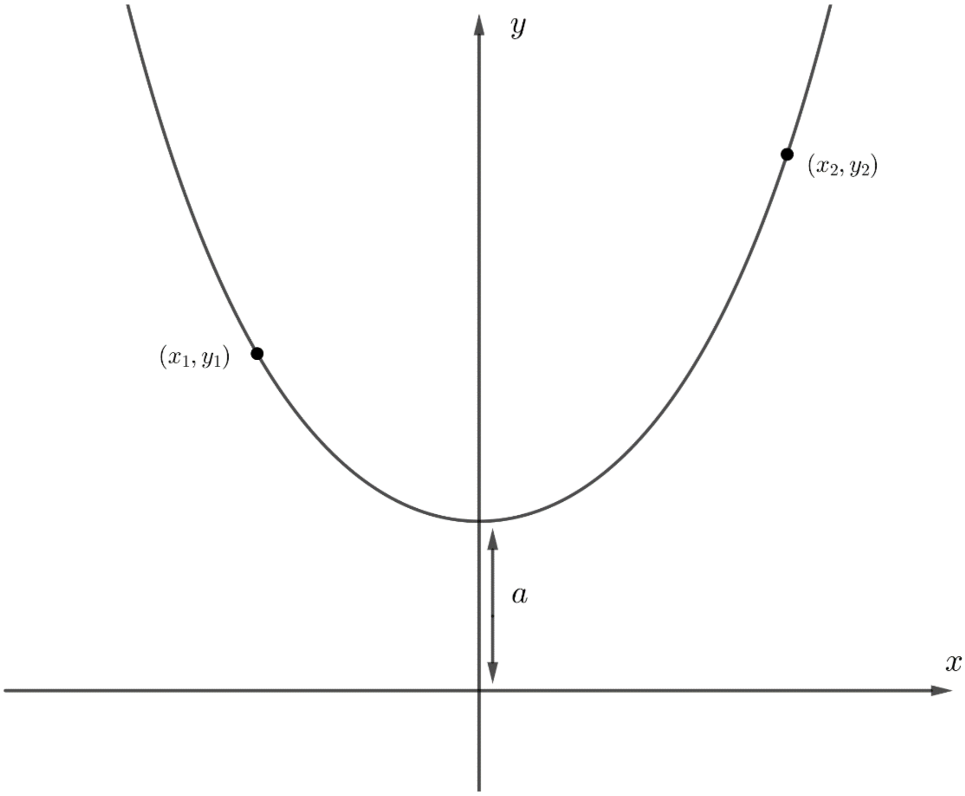

Figure S1 is showing a catenary which is given by the equation:

$$y = a\cosh\left(\frac{x}{a}\right) \qquad [1]$$

The arclength of the curve $l_t = l_1 + l_2$ along the catenary is given by the equation between the points (x_1, y_1) and (x_2, y_2):

$$l_t = l_1 + l_2 = \int_{x_1}^{x_2} \sqrt{1 + \left(\frac{dy}{dx}\right)^2}\, dx = a\sinh\left(\frac{x_2 - x_1}{a}\right) \qquad [2]$$

$$l_1 = a\sinh\left(\frac{x_1}{a}\right) \qquad [3]$$

$$l_2 = a\sinh\left(\frac{x_2}{a}\right) \qquad [4]$$

Using the hyperbolic functions relationship, equations [1] and [2] can be related as follows:

$$\sqrt{l_t^2 + (y_2 - y_1)^2} = 2a\sinh\left(\frac{x_2 - x_1}{2a}\right) \qquad [5]$$

In catenary equation, $a = \frac{T_0}{w}$ where $T_0$is the horizontal component of the tension at the point that cable is hanging and w is the weight per unit length of the cable. These values can be assumed to be constant in the problem we are trying to solve.

Mathematics of a circle:

Figure S2

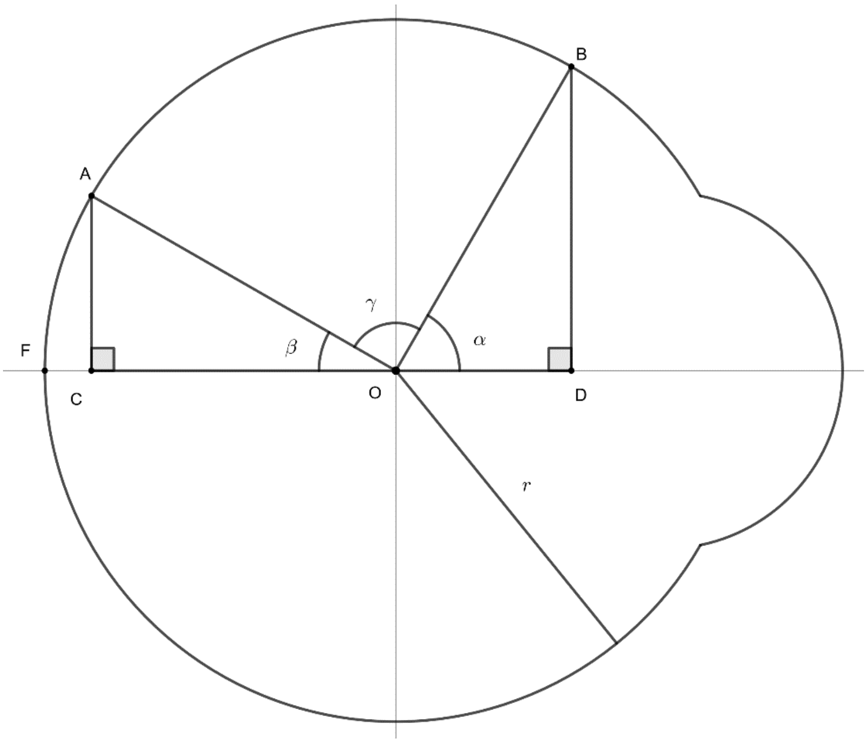

The inner globe can be treated as a circle with centre O and radius r as shown in Figure S2.

The following are given to us in relation to Figure S2:

The inner radius of the globe is $\overline{FO} = r = 11mm$ (1)

The ora serrata, point B, makes an angle $\alpha = 60° = \frac{\pi}{3}$ with the horizontal (1)

Therefore, $\beta + \gamma = 120° = \frac{2\pi}{3}$

The arc $AB = \gamma r$, arc $FA = \beta r$, and arc $FB = (\beta + \gamma)r$ where angles γ and β are measured in radians

$\overline{BD} = r \sin \alpha$, $\overline{OD} = r \cos \alpha$, $\overline{AC} = r \sin \beta$, $\overline{OC} = r \cos \beta$, and $\overline{FC} = r - r \cos \beta$

Scenario 1:

In this scenario the RRD represented by the catenary arc FB extends from the ora serrata at point B to fovea at point F. We would like to calculate how much the trough of the RRD hangs below the visual axis given by $y_1 - a$ (Figure S3).

Figure S3:

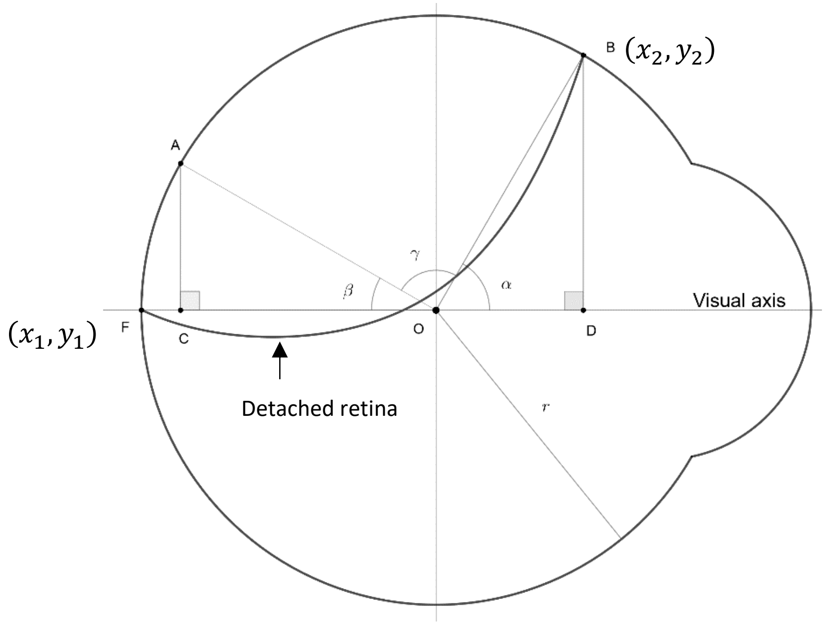

In order to solve the problem, we need to calculate the value of the constant a.

The arc FB of the circle and the arclength l_t of the catenary are equal and therefore:

$$l_t = FB = (\beta + \gamma)r = \frac{2\pi}{3} \cdot 11$$

And

$$y_2 - y_1 = \overline{BD} = r \sin \alpha = 11 \sin \frac{\pi}{3}$$

$$x_2 - x_1 = \overline{FO} + \overline{OD} = r + r \cos \alpha = 11 + 11 \cos \frac{\pi}{3}$$

Inserting these values in the equation [5],

$$\sqrt{\left(\frac{2\pi}{3} \cdot 11\right)^2 + \left(11 \sin \frac{\pi}{3}\right)^2} = 2a \sinh\left(\frac{11 + 11 \cos \frac{\pi}{3}}{2a}\right)$$

With aid of Python Version 3.6 and 'math' module, we used trial and error method of iteration and calculated constant a as $a = 6.714439009374067$.

Next we need to calculate the coordinates of points (x_1, y_1) and (x_2, y_2) for this catenary.

$$l_t = l_1 + l_2$$

$$x_2 = (x_2 - x_1) + x_1$$

Therefore,

$$a \sinh\left(\frac{x_2 - x_1}{a}\right) = a \sinh\left(\frac{x_1}{a}\right) - a \sinh\left(\frac{(x_2 - x_1) + x_1}{a}\right)$$

$$\frac{2\pi}{3}\cdot 11 = 6.7\ldots \sinh\left(\frac{x_1}{6.7\ldots}\right) - 6.7\ldots \sinh\left(\frac{\left(11 + 11\cos\frac{\pi}{3}\right) + x_1}{6.7\ldots}\right)$$

And using the iteration method above long with equations [1] to [5], the coordinates were calculated as below:

$$x_1 = -5.30mm$$

$$x_2 = 11.20mm$$

$$y_1 = 8.91mm$$

$$y_2 = 18.44mm$$

Therefore, the distance of the trough of the curve below the visual axis is given by:

$$y_1 - a = 8.91 - 6.71 = 2.20mm$$

Scenario 2:

In this scenario the RRD represented by the catenary arc AB extends from the ora serrata at point B to point A as shown in Figure S4. We would like to calculate the length of the arc FA which is given by $FA = r\beta$ in Figure S4.

Figure S4:

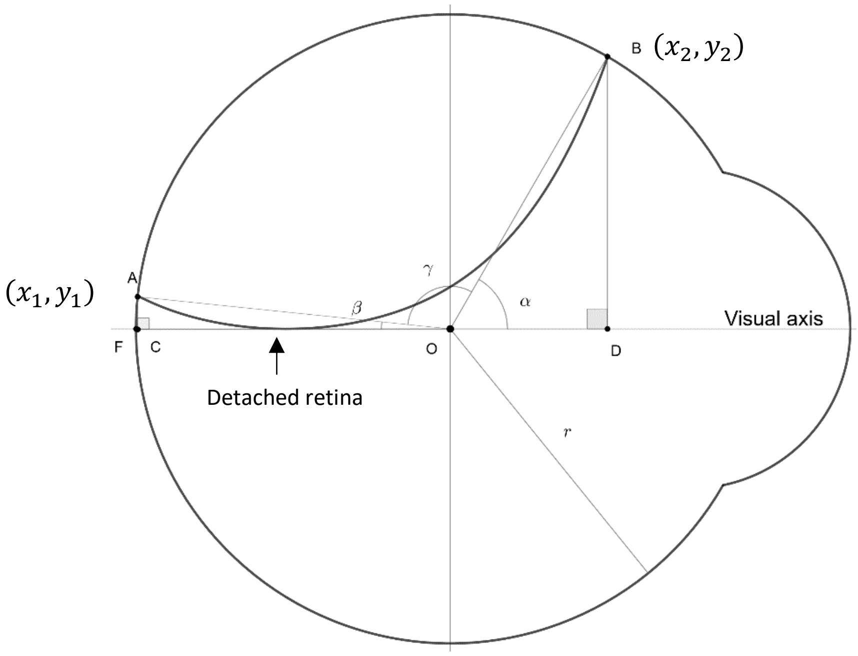

The value of y_2 is known and is given by:

$$y_2 = \overline{BD} + a = r\sin\alpha + a = 11\sin\frac{\pi}{3} + 6.7\ldots = 16.24\ldots$$

Using y_2, x_2 can be calculated as follows:

$$x_2 = a\cosh^{-1}\left(\frac{y_2}{a}\right) = 6.7\ldots\cosh^{-1}\left(\frac{16.2\ldots}{6.7\ldots}\right) = 10.27\ldots$$

The constant a can be written in term of y_1, r and β:

$$y_1 - a = r\sin\beta \Rightarrow a = y_1 - 11\sin\beta = a\cosh\left(\frac{x_1}{a}\right) - 11\sin\beta \qquad [6]$$

We can calculate $x_1$as follows:

$$|x_1| + |x_2| = r\cos\beta + r\cos\alpha = 11\cos\beta + 11\cos\left(\frac{\pi}{3}\right)$$

$$|x_1| = 11\cos\beta + 11\cos\left(\frac{\pi}{3}\right) - 10.27\ .... \qquad [7]$$

Combining the equations [6] and [7] to calculate the angle β:

$$a = a\cosh\left(\frac{11\cos\beta + 11\cos\left(\frac{\pi}{3}\right) - 10.27\ ...}{a}\right) - 11\sin\beta$$

$$6.7\ ... = 6.7\ ...\cosh\left(\frac{11\cos\beta + 11\cos\left(\frac{\pi}{3}\right) - 10.27\ ...}{6.7..}\right) - 11\sin\beta$$

Using the iterative method, $\beta = 0.25169136928676034\ radians$

Therefore,

$$arc\ FA = r\beta = 11 \times 0.251\ ... = 2.768\ ...$$

Therefore, when the trough of the RRD just touches the visual axis and starts to obscure the fovea, the closest edged of the RRD to fovea is 2.77mm or 14.4° away from the fovea.

Scenario 3:

Figure S5

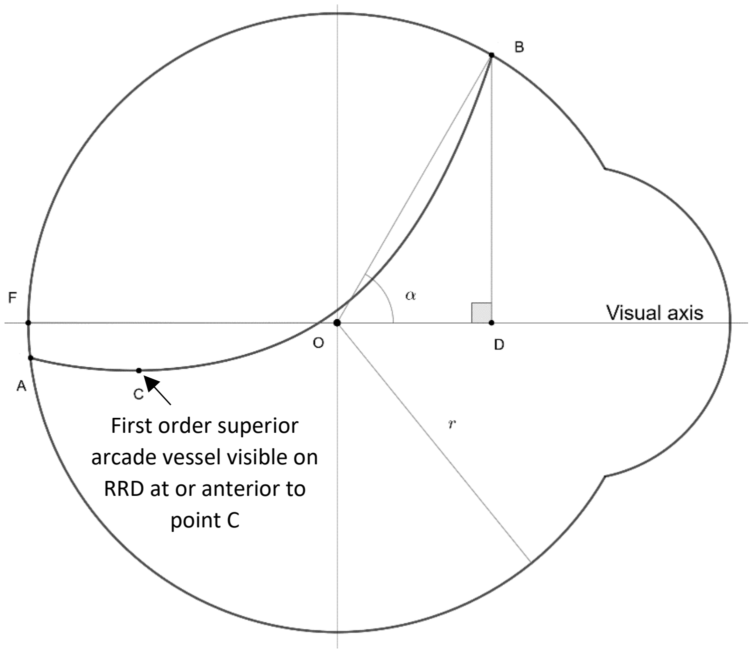

We would like to ascertain the position of the point A on the Figure S5 when the first order superior arcade vessel is visible on the RRD at the its trough (point C).

Let's assume that the first order superior arcade vessel is $15°$ or $\frac{\pi}{12}$ above the fovea. This means the arclength CB is given by,

$$CB = \frac{7\pi}{12} = \frac{77\pi}{12} = a\sinh\frac{x}{a}$$

where x is the horizontal distance between points B and C. The value of x can be calculated as follows,

$$x = a\sinh^{-1}\left(\frac{77\pi}{12a}\right)$$

$$x = 6.7\ldots\sinh^{-1}\left(\frac{77\pi}{12\times 6.7\ldots}\right) = 12.24737500590527mm$$

Therefore, the horizontal distance between points A and C ($\overline{AC}$) is very close to,

$$\overline{AC} \approx r + r\cos\alpha - x = 11 + 11\cos\frac{\pi}{3} - 12.2\ldots = 4.285335803611268mm$$

The arclength AC can be calculated as follows,

$$arclength\ AC \approx a\sinh\frac{\overline{AC}}{a} \approx 4.582245698093238mm$$

We can calculate the distance between points A and B on the circle in angles

$$\frac{(arclength\ AC + arclength\ BC)}{r} = \frac{\left(4.5\ldots + \frac{77\pi}{12}\right)}{11} = 2.249163505329795\ radians$$

Therefore the distance between points F and A on the circle in angle is

$$2.249163505329795 - \frac{2\pi}{3} = 0.15476840293659988\ radians$$

Therefore, if the first order superior arcade vessel is visible on the RRD, even if it is at the trough of the RRD, the edge of the RRD is 8.8° or $1.7mm$ below the fovea – i.e. the fovea is detached.

<u>Scenario 4:</u>

Figure S6

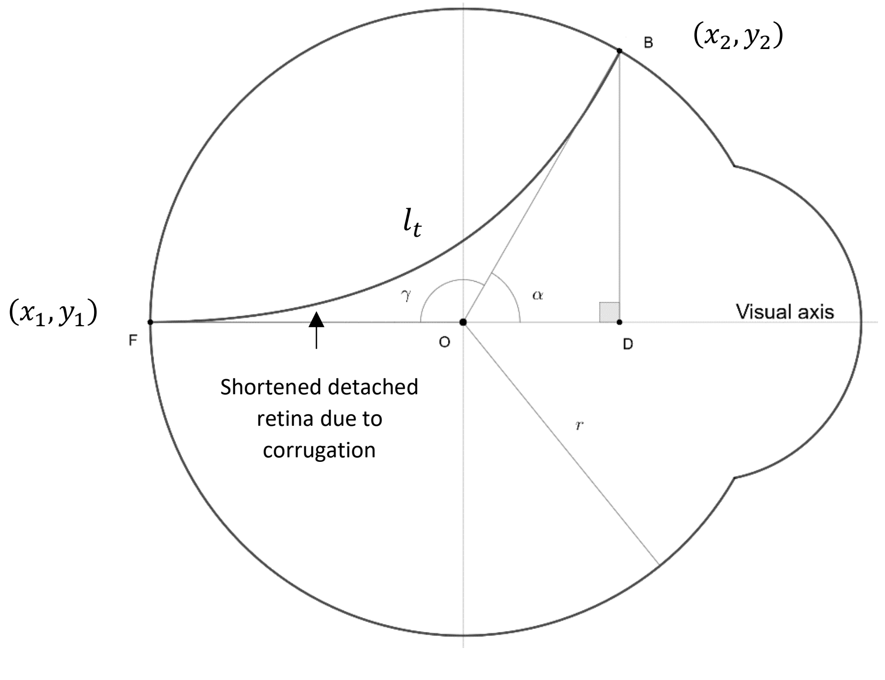

If the RRD is starting from the ora serrata and extends to the fovea, how much the retina needs to be shortened by the corrugation before the trough of the RRD is at the level of the fovea on the visual axis, because the retina is lifted, as illustrated in Figure S6. Therefore, we need to calculate the value of l_t in this scenario.

In this situation, we know that,

$$y_2 - y_1 = r \sin \alpha = 11 \sin\left(\frac{\pi}{3}\right)$$

Also,

$$y_2 - y_1 = a \cosh\left(\frac{x_2 - x_1}{a}\right) - a \qquad [8]$$

And the hyperbolic identity,

$$(\cosh \theta)^2 - (\sinh \theta)^2 = 1 \qquad [9]$$

Using equations [2], [8] and [9],

$$\left(\frac{(y_2 - y_1) + a}{a}\right)^2 - \left(\frac{l_t}{a}\right)^2 = 1$$

$$\left(\frac{l_t}{a}\right)^2 = \left(\frac{\left(11 \sin\left(\frac{\pi}{3}\right)\right) + a}{a}\right)^2 - 1$$

$$l_t = a\left(\sqrt{\left(\frac{\left(11 \sin\left(\frac{\pi}{3}\right)\right) + a}{a}\right)^2 - 1}\right)$$

Inserting the value of $a = 6.714439009374067$

$$l_t = 6.7 \ldots\left(\sqrt{\left(\frac{\left(11 \sin\left(\frac{\pi}{3}\right)\right) + 6.7 \ldots}{6.7 \ldots}\right)^2 - 1}\right)$$

$$l_t = 14.78773967157055$$

Therefore, the retina is shortened by,

$$r\gamma - l_t = 11 \times \frac{2\pi}{3} - 14.7 \ldots = 8.250606454754598$$

Which is $8.25mm$.

<u>Myopic eye as an ellipsoid:</u>

The dimensions of the myopic eye was taken from the study by Pope et al (2). For a -10 dioptre myopic eye, the height was calculated to be $25.13mm$ and length was calculated to be $27.13mm$. Therefore, the sagittal section of a myopic eye is an ellipse (Figure S7).

The length of the detached retinal arc for a myopic eye can be calculated using the equation for ellipse:

$$\frac{x^2}{a^2} + \frac{y^2}{b^2} = 1 \; where \; a = \frac{length}{2} \; and \; b = \frac{height}{2}$$

Also, the equation of the ellipse in parametric form:

$$x = a\cos\theta, y = b\sin\theta$$

The length of the retinal arc on an ellipse extending from ora serrata to the fovea, S, can be calculated by obtaining the value of the following integral with *SciPy*.

$$S = a\left[\int_0^{\frac{\pi}{2}} \sqrt{1 - (1 - \frac{b^2}{a^2})\sin^2\theta \, d\theta} + \int_\theta^{\frac{\pi}{2}} \sqrt{1 - (1 - \frac{b^2}{a^2})\sin^2\theta \, d\theta}\right]$$

Figure S7:

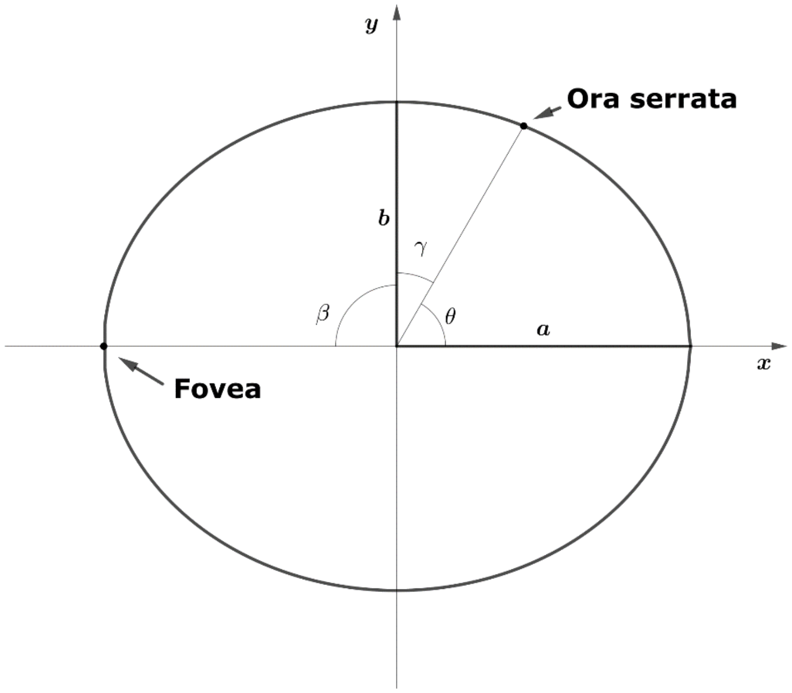

The scenarios 1 and 2 for the myopic eye was calculated as described for emmetropic eye. However, we calculated the x and y coordinates and arclength for an ellipse as described above.

Reference:

1. Taylor E, Jennings A. Calculation of total retinal area. Br J Ophthalmol. 1971;55(4):262-5.
2. Pope JM, Verkicharla PK, Sepehrband F, Suheimat M, Schmid KL, Atchison DA. Three-dimensional MRI study of the relationship between eye dimensions, retinal shape and myopia. Biomed Opt Express. 2017;8(5):2386-95.
